# Perceptions of Using Instant Messaging Apps for Alcohol Reduction Intervention Among University Student Drinkers: Semistructured Interview Study With Chinese University Students in Hong Kong

**DOI:** 10.2196/40207

**Published:** 2023-02-27

**Authors:** Siu Long Chau, Yiu Cheong Wong, Ying Pei Zeng, Jung Jae Lee, Man Ping Wang

**Affiliations:** 1 School of Nursing The University of Hong Kong Hong Kong Hong Kong

**Keywords:** instant messaging apps, mobile phone, WhatsApp, alcohol reduction intervention, alcohol use, university students, young adults, instant messaging, alcohol reduction, adverse lifestyle, intervention, health promotion, text messages, health behaviours, health behaviors, apps

## Abstract

**Background:**

Mobile instant messaging (IM) apps (eg, WhatsApp and WeChat) have been widely used by the general population and are more interactive than text-based programs (SMS text messaging) to modify unhealthy lifestyles. Little is known about IM app use for health promotion, including alcohol reduction for university students.

**Objective:**

This study aims to explore university student drinkers' perceptions of using IM apps for alcohol reduction as they had high alcohol exposure (eg, drinking invitations from peers and alcohol promotion on campus) and the proportion of IM app use in Hong Kong.

**Methods:**

A qualitative study was conducted with 20 Hong Kong Chinese university students (current drinkers) with Alcohol Use Disorder Identification test scores of ≥8 recruited using purposive sampling. Semistructured individual interviews were conducted from September to October 2019. Interview questions focused on drinking behaviors, quitting history, opinions toward IM app use as an intervention tool, perceived usefulness of IM apps for alcohol reduction, and opinions on the content and design of IM apps for alcohol reduction. Each interview lasted approximately 1 hour. All interviews were audio-taped and transcribed verbatim. Two researchers independently analyzed the transcripts using thematic analysis with an additional investigator to verify the consistency of the coding.

**Results:**

Participants considered IM apps a feasible and acceptable platform for alcohol reduction intervention. They preferred to receive IMs based on personalized problem-solving and drinking consequences with credible sources. Other perceived important components of instant messages included providing psychosocial support in time and setting goals with participants to reduce drinking. They further provided suggestions on the designs of IM interventions, in which they preferred simple and concise messages, chat styles based on participants' preferences (eg, adding personalized emojis and stickers in the chat), and peers as counselors.

**Conclusions:**

Qualitative interviews with Chinese university student drinkers showed high acceptability, engagement, and perceived utility of IM apps for alcohol reduction intervention. IM intervention can be an alternative for alcohol reduction intervention apart from traditional text-based programs. The study has implications for developing the IM intervention for other unhealthy behaviors and highlights important topics that warrant future research, including substance use and physical inactivity.

**Trial Registration:**

ClinicalTrials.gov NCT04025151; https://clinicaltrials.gov/ct2/show/NCT04025151?term=NCT04025151

## Introduction

Alcohol use is one of the leading risk factors for premature death and disability-adjusted life year loss (DALY) in young adults (aged 18-25 years) [1]. The prevalence of alcohol use and binge drinking (>5 standard drinks for a male and >4 standard drinks for a female on one occasion, one standard drink containing 14 g of alcohol) among Hong Kong (HK) young adults had doubled in a decade [2]. University students had a high level of alcohol exposure (eg, drinking invitations from peers and alcohol promotion on campus) and alcohol consumption [3]. Alcohol misuse contributed to various risky behaviors, such as substance use, unsafe sex, school absenteeism, and physical violence in university students [4].

As communication technologies advanced, mobile phones provided a new avenue to deliver behavioral change intervention [5]. Evidence showed that SMS text messaging was effective in changing adverse lifestyles [6]. A systematic review of 9 randomized controlled trials showed that using SMS text messaging to deliver brief alcohol interventions (evidence-based intervention developed by the World Health Organization to reduce alcohol consumption) reduced the weekly alcohol unit consumption by 13 g per week in university students [7,8]. SMS text messages can be personalized in accordance with the drinkers' characteristics and allow recipients to text keywords to receive on-demand support [8]. Nevertheless, SMS text messages have several limitations, including text-only communication, limited character count, high costs per message, and low interaction with participants [7]. Although instant messaging (IM) apps are widely used by university students, their acceptability and perceptions of using IM apps for alcohol reduction remain unclear.

IM apps (eg, WhatsApp, Signal, and WeChat) provide more alternative modes of communication. For example, it allows video chats, voice calls, and the exchange of pictures and animation with participants, which overcome most of the limitations of SMS text messaging and have been widely used by the general population [5]. Our studies found that IM apps are effective in promoting healthier lifestyles, including helping participants to engage in more physical activities and achieve a higher rate of smoking abstinence than the control group that received the intervention via SMS text message [9,10]. Studies found that IM apps are effective in preventing smoking relapse in people who have quit smoking cigarettes and promoting healthy eating in participants with obesity in randomized controlled trials [10,11]. Emerging research studied integrating IM into health care but primarily focused on using IM to promote clinical patient management (eg, weight management) and interprofessional communication (eg, telemedicine) [11,12]. The use of IM apps for alcohol reduction in university students has remained uncertain.

HK is the most westernized city in China and has zero tax on liquor with an alcoholic percentage of <30% [2]. Novel interventions are needed to stop the growing trend of alcohol misuse among university students. Leveraging the extensive smartphone penetration in HK (88.6% in 2017) [13], IM apps may provide better prevention and treatment for alcohol misuse. As no research studied IM apps to deliver alcohol reduction intervention among university students, we conducted a formative qualitative study to inform the designs and contents of the IM intervention. We also aim to explore the perceptions of using IM intervention for alcohol reduction in HK university students.

## Methods

### Study Design

We recruited university students from 3 local universities using purposive sampling in HK and used semistructured, individual interviews. The eligibility criteria included (1) being a HK resident aged ≥18 years, (2) studying at a local university, (3) being able to communicate in Cantonese, (4) drinking alcohol at least monthly (ie, current drinker), and (4) using a smartphone with installed IM apps. Participants with a history of psychiatric disorders or those participating in any alcohol treatment program were excluded. We recruited participants through universities' mass emails, posters, and social media (eg, Instagram and Facebook) with embedded QR codes. The QR code directed the students to the baseline questionnaire. Eligible participants who filled out the baseline questionnaire were invited to participate in the individual interview. To increase heterogeneity, we selected participants of different sex, years of study, and drinking patterns at baseline. Recruitment ceased after achieving information redundancy (no new information emerged).

### Ethical Considerations

The study was approved by the institutional review board of the University of Hong Kong/Hospital Authority Hong Kong West Cluster (UW 19-103). Individual interviews were conducted in a confidential area. We obtained verbal and written informed consent from all participants before the interview began. The institutional review board also allowed secondary analysis without additional consent. This study strictly followed the Consolidated Criteria for Reporting Qualitative Research guidelines [14]. A coupon of HK $100 (US $12.8) was provided to the participants as an incentive.

### Data Collection

We interviewed 20 university students from September to October 2019. Each interview lasted approximately 1 hour. All interviews were conducted in a meeting room at the University of Hong Kong. Participants completed a web-based baseline questionnaire on their sociodemographic and drinking characteristics before being invited for the interview.

A research nurse (SLC) conducted the individual interviews. Only the research nurse and interviewee were present throughout the interview. The discussion began with the interviewer asking the participants to describe their perception of drinking, cues of drinking, and previous quit attempts as opening questions. Following the interview guide (available in Multimedia Appendix 1), the interviewer asked open-ended questions (eg, “What do you think about using mobile instant messaging apps for giving drinking advice to university students?” and “What would strengthen your motivation to quit or reduce drinking?”). All interviews were conducted in Cantonese and audio-taped with participants' consent. A HK $100 (US $12.78) cash coupon was given to each participant as a study participation incentive. No repeated interviews were carried out.

### Data Analysis

The audio-taped interviews were transcribed verbatim. Interim analyses were performed after each interview to refine the interview questions for in-depth understanding. Two researchers (YCW and YPZ) analyzed the transcripts using thematic analysis [15]. The researchers first read all the transcripts to familiarize themselves with the data and generate initial thoughts on the data. All the passages referred to the research question were coded, and codes that shared similar meanings were grouped into subcategories. The codes were then reviewed and organized into themes. Disagreements in coding were resolved through discussion with an additional researcher (MPW), who further reviewed and verified the consistency of the coding [16]. Interview data and coding were managed using NVivo (version 12; QSR International). Selected interview excerpts were translated into English and back-translated by researchers who are bilinguals for reporting. Information redundancy for data analysis was achieved at 20 participants as no new information was identified.

### Rigor

Rigor was assured by following the systematic and step-by-step process of thematic analysis guidelines strictly [16]. Data collection and analysis stages were undertaken concurrently, and an interim analysis was performed after each interview to ensure that the developing themes were grounded in the original data [16].

## Results

### Sample Characteristics

Table 1 shows that most participants were female (13/20, 65%), studying in their first to third year of university (13/20, 65%), had no previous quit attempt (11/20, 55%), and had no intention to quit (12/20, 60%). Further, 90% (18/20) and 85% (17/20) of participants received and sent more than 21 instant messages per day, respectively.

We identified 2 major themes from the data by thematic analysis. These included (1) the perceived important components of the IM intervention for alcohol reduction and (2) suggestions for designing the proposed IM intervention.

**Table 1:** Sample characteristics of the participants (N=20).

Characteristics			Participants, n (%)
**Sex**			
	Male	7 (35)	
	Female	13 (65)	
**Year of study^a^**			
	1-3	13 (65)	
	4-5	7 (35)	
**Perceived family affluence**			
	Below average	10 (50)	
	Average	9 (45)	
	Above average	1 (5)	
**Previous quit attempts**			
	0	11 (55)	
	1	3 (15)	
	>2	6 (30)	
**Intention to quit**			
	Quit within 1 month	2 (10)	
	Quit between 1 to 6 months	6 (30)	
	Not planning to quit	12 (60)	
**Number of instant messages received per day**			
	1-10	1 (5)	
	11-20	1 (5)	
	21-50	7 (35)	
	51-100	4 (20)	
	>100	7 (35)	
**Number of instant messages sent per day**			
	1-10	1 (5)	
	11-20	2 (10)	
	21-50	7 (35)	
	51-100	7 (35)	
	>100	3 (15)	

^a^Years 1, 2, 3, and 4 are equivalent to freshman (aged 18 years), sophomore (aged 19 years), junior (aged 20 years), and senior (aged 21 years) year in the North American university system.

### Theme 1: Perceived Important Components of the IM Intervention for Alcohol Reduction

Participants considered using an IM app as a feasible and acceptable platform for providing alcohol reduction intervention. They preferred an IM app to other platforms (eg, email, Facebook, and Instagram) as it allowed for real-time interaction with the counselor and was able to provide drinking advice when needed.

Email is boring and sometimes too lengthy to read. Sending messages by WhatsApp is more direct and much easier. You can interact with the counselor using IM apps anytime and anywhere you want, which cannot be achieved by email or other means.

#8, female, year 2

We further identified 4 subthemes on the type of messages that the participants wish to receive. They preferred to receive instant messages based on personalized problem-solving, providing drinking consequences with credible sources, providing psychosocial support in time, and setting drinking goals with participants.

### Personalized Problem-solving

Participants perceived IM apps as a useful platform to obtain information about quitting and to inquire about personalized drinking advice when needed.

I think you can provide more personalized feedback based on their characteristics, like sex. For example, females may care more about their appearance, and you can tell how drinking affects their skin. I would be attracted to that information.

#20, female, year 2

The importance of providing in-time drinking advice was further highlighted by the participants.

I always go out with my friend to have drinks on Friday night and end up drunk every time. It would be helpful if you could immediately give me some drinking advice to ease my drunkenness.

#4, female, year 4

### Providing Drinking Consequences With Credible Sources

Regular receipt of messages related to negative consequences of drinking was considered by participants as a persuasive motivation to quit drinking. They indicated that these messages would create a daunting effect and encourage them to monitor their drinking amount.

I have gained several pounds these years because I have been drinking a lot of beers to reduce my academic stress. If you can show me more scientific evidence of the harm of drinking, I will definitely start to control my drinking amount.

#2, female, year 4

### Providing Psychosocial Support in Time

Participants considered drinking a way to cope with stress, such as academic pressure. They would appreciate if the counselor could provide timely psychological support via an IM app to facilitate their quitting process.

I want to quit drinking but have found it difficult as all my friends drink. I think having someone encouraging me can push me harder to stop drinking.

#20, female, year 2

Participants further suggested that simple messages, such as appreciating the effort of quitting, will motivate them to stay on the right track.

Quitting is not easy. I drank a lot again because no one even appreciate my quitting efforts. I need someone who can acknowledge my hard work and support my quitting.

#6, male, year 3

### Setting Drinking Goals With Participants

Participants highlighted the importance of having a sense of achievement from quitting. They suggested that the counselor personalize the objective and monitor each participant's progress regularly, which can strengthen their commitment to quitting.

I think you can set a target, like first cutting down my weekly alcohol consumption from 5 bottles to 3 bottles of beer and then checking my progress in WhatsApp. This would motivate me to quit.

#16, female, year 1

### Theme 2: Suggestions for Designing the Proposed Intervention

Participants provided suggestions on the designs and methods for delivering the proposed intervention. These suggestions included (1) optimal text length and delivery frequency, (2) adding interesting elements in the chat, and (3) peer as a counselor.

### Optimal Text Length and Delivery Frequency

When asked about the design of the intervention, participants proposed that the messages should be simple and concise. They also preferred not to receive messages every day. They referred lengthy and frequent messages as aversive and not necessary.

I prefer to keep the messages short and precise. If you send me a long message every day, I will find it annoying and just delete it.

#10, male, year 1

Participants further suggested sending the messages specifically on weekends as they may have more time to drink.

I don't have much time to drink from Monday to Friday due to schoolwork and part-time. It may be helpful if can remind me on the weekends, like Saturday night.

#20, female, year 2

### Adding Interesting Elements in the Chat

Participants preferred adding more personalized emojis or stickers in the chat, which would create a more casual and closer relationship with the counselor. Some recommended adding specific emojis relevant to the texts, and they described these messages as more noticeable.

I think adding relevant emoji or stickers in the texts can make the messages more eye-catching. For example, when you are talking about the harm of drinking in the text, you can add a “skull” emoji next to it. People will immediately understand alcohol is bad for health.

#10, male, year 1

Other participants preferred to receive pictures or animation besides written language. They believed the media would be a convincing way to reinforce the idea of quitting.

Videos, especially cartoon animation about the harm of drinking and the benefits of quitting, sound more impressive and credible compared with only written language.

#8, female, year 1

### Peer as a Counselor

Some participants suggested that having a peer as a counselor to give drinking advice would help strengthen the intervention. They believed that peers could better understand their drinking history and empathize with their challenges while quitting.

Talking to someone of similar age would be easier to communicate, as we may have similar struggles in life.

#12, male, year 3

## Discussion

### Principal Findings

This study explored the perceptions of using IM apps for alcohol reduction intervention in Hong Kong university students who speak Chinese or are of Chinese origin. Participants considered an IM app as a feasible and acceptable platform for delivering drinking advice. Based on their preference, they preferred to receive IMs based on personalized problem-solving (eg, identify ways to stop drinking), drinking consequences with credible sources (eg, provide scientific evidence on the harm of drinking), in-time psychosocial support (eg, address emotional needs promptly), and goal setting to reduce drinking (eg, personalized drinking goals) [17]. They also provided suggestions on the designs of the proposed intervention, which further improved its acceptability and feasibility.

Participants considered the ability to provide in-time drinking advice to them as one of the primary merits of using IM apps. A similar finding was obtained in another formative study of developing a text messaging–based intervention for alcohol reduction in the United States [18]. Evidence suggested that immediate informational and psychological support were important to enhance confidence to quit drinking; it facilitated quitting by helping the drinkers tackle challenges in time and promoted their motivation to quit [19]. In addition, clinical consultation with health care professionals was limited by time constraints and sometimes could be very brief. By using IM apps, counselors can learn about their quitting process regularly, tailor specific interventions that suit their drinking characteristics, and facilitate quitting more effectively.

Apart from addressing personal needs, the ability to provide credible information on the harm of drinking was regarded by participants as a crucial motivator to reduce drinking. Unlike mobile health trials of smoking cessation, smokers preferred receiving gain-framed messages (eg, benefits of quitting smoking) to loss-framed messages (eg, harm of smoking) as they were already aware of the harm of smoking [20]. Emerging research showed that drinkers had misconceptions about alcohol use and considered it beneficial to health (eg, drinking low to moderate red wine can reduce the risk of cardiovascular diseases) [21]. Providing credible information on drinking harm clarifies their fallacy of alcohol and promotes their motivation to quit.

Participants emphasized the importance of receiving emotional support from the counselor during the process of quitting when using IM apps. A study showed that in-time person-to-person psychosocial support was one of the important factors for successful quitting in substance use treatment, as sustained external emotional support was a key mediator for achieving long-term abstinence [22]. By using IM apps, counselors can determine drinkers' emotional responses to relapse while quitting and promptly tailor the appropriate intervention to address their emotional needs [22]. Compared with traditional text-based interventions (eg, SMS text messaging), the proposed intervention allows real-time interaction with the counselor and facilitates better quitting outcomes by enhancing drinkers' psychosocial supports.

Apart from using IM apps for providing alcohol reduction intervention, the proposed intervention may also inform the design of similar interventions for treating other adverse health behaviors, including using IM apps for substance use treatment. The IM intervention would need counselors to deliver a chat messaging intervention to participants, which may incur higher operational costs than conventional text messaging–based interventions. Nevertheless, as mobile IM apps are freely available in the market and are widely used by the general population, and artificial intelligence–based technologies are becoming more advanced and mature, the development of chatbots would reduce the operational cost of IM interventions in the future [23].

### Limitations

This study has some limitations. First, as it focused on using IM apps for alcohol reduction intervention, non–smartphone owners were excluded. The views expressed during the interview were specific to university student drinkers who own a smartphone; they are typically younger and have higher digital literacy than the general population [13]. Second, the representativeness of the study was limited as participants were recruited from universities in Hong Kong, and over half of the participants (60%) had no intention to quit drinking; hence, our findings may not be generalizable to non-Chinese university students and people who have the intention to quit or reduce drinking. Third, member checking was not conducted due to lack of funding; we improved the trustworthiness of the findings by having 2 researchers independently code the transcripts and an additional investigator to further verify the consistency and accuracy of the coding.

### Conclusions

This formative study has informed the designs and contents of an IM intervention for alcohol reduction in university student drinkers. It provides an alternative to traditional SMS text messaging–based intervention. The proposed intervention will be evaluated in a randomized controlled trial with Hong Kong university settings (NCT04025151).

## Appendix

Multimedia Appendix 1 Semistructured interview guide.

## Abbreviations

DALY: disability-adjusted life year loss
HK: Hong Kong
IM: instant messaging

## Data Availability

The data set generated during and/or analyzed during the current study is available from the corresponding author upon reasonable request.
