# Supplementary material for: Perceptions of Using Instant Messaging Apps for Alcohol Reduction Intervention Among University Student Drinkers: Semistructured Interview Study With Chinese University Students in Hong Kong
Source: JMIR Form Res. 2023 Feb 27;7:e40207. doi: 10.2196/40207 (PMC10012002; doi:10.2196/40207)
Supplement: Multimedia Appendix 1 [file formative_v7i1e40207_app1.doc]

|  |  |
| --- | --- |

**Semi-structured interview guide**

1. Perceptions of drinking alcohol
2. How do you feel about drinking alcohol as a university student?
3. What kinds of activities or social cues in your environment will urge you to drink?
4. Could you share your experience of previous quit attempts or ways to control the drinking amount?
5. Perceptions of using mobile instant messaging (IM) apps
6. How do you feel about the mobile instant messaging (IM) apps nowadays?
7. What do you think about using mobile IM apps for giving drinking advice to university students?
8. What would strengthen your motivation to quit or reduce drinking?
